# Analysis of the spatial distribution of infant mortality by cause of death in Austria in 1984 to 2006

**DOI:** 10.1186/1476-072X-7-21

**Published:** 2008-05-21

**Authors:** Thomas Waldhoer, Martin Wald, Harald Heinzl

**Affiliations:** 1 Center of Public Health, Department of Epidemiology, Medical University of Vienna, Borschkegasse 8a, 1090 Vienna, Austria; 2 Division of Neonatology and Intensive Care, Department of Pediatrics, Medical University of Vienna, Austria; 3 Section of Clinical Biometrics, Core Unit for Medical Statistics and Informatics, Medical University of Vienna, 1090 Vienna, Austria

## Abstract

**Background:**

In Austria, over the last 20 years infant mortality declined from 11.2 per 1,000 life births (1985) to 4.7 per 1,000 in1997 but remained rather constant since then. In addition to this time trend we already reported a non-random spatial distribution of infant mortality rates in a recent study covering the time period 1984 to 2002.

This present study includes four additional years and now covers about 1.9 million individual birth certificates. It aimes to elucidate the observed non-random spatial distribution in more detail. We split up infant mortality into six groups according to the underlying cause of death. The underlying spatial distribution of standardized mortality ratios (SMR) is estimated by univariate models as well as by two models incorporating all six groups simultaneously.

**Results:**

We observe strong correlations between the individual spatial patterns of SMR's except for "Sudden Infant Death Syndrome" and to some extent for "Peripartal Problems". The spatial distribution of SMR's is non-random with an area of decreased risk in the South-East of Austria. The group "Sudden Infant Death Syndrome" clearly and the group "Peripartal Problems" slightly show deviations from the common pattern. When comparing univariate and multivariate SMR estimates we observe that the resulting spatial distributions are very similar.

**Conclusion:**

We observe different non-random spatial distributions of infant mortality rates when grouped by cause of death. The models applied were based on individual data thereby avoiding ecological regression bias. The estimated spatial distributions do not substantially depend on the employed estimation method. The observed non-random spatial patterns of Austrian infant mortality remain to appear ambiguous.

## Background

**Figure 1:**
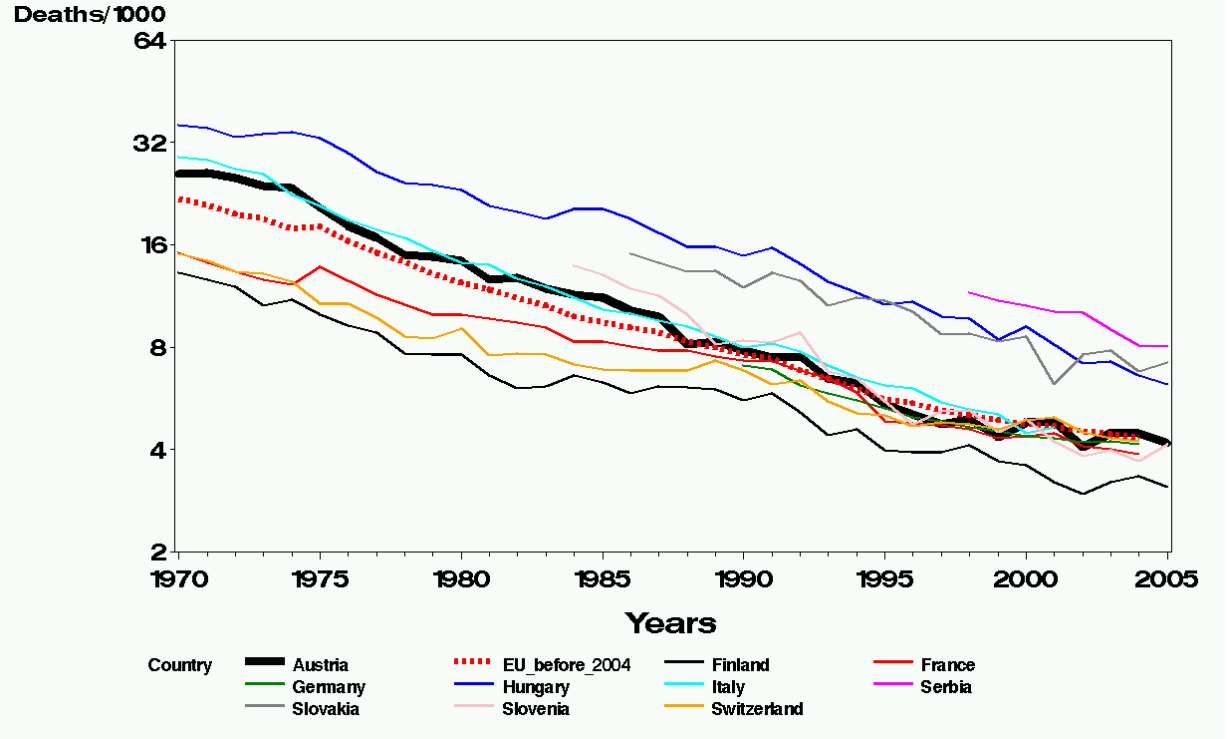
Infant mortality rates per 1000 life births for some European countries.

Infant mortality rate in Austria was higher than average in the European Union until 1987 but now has reached the European Union means [1,2] (Figure 1). Despite this very welcome temporal trend, in a recent study [3] including the years 1984–2002, we observed an explicit non-uniform spatial distribution indicating lower risks in the South-East of Austria. This study was based on about 1.6 million individual birth certificates allowing the adjustment of the infant mortality rates for a large number of covariates. The then observed non-uniform spatial distribution was surprising because the covariates comprised many important anthropometric as well as socio-economic factors. To our knowledge, these covariates covered the most important prognostic factors, so only minor systematic differences in infant mortality rates due to variables not included in the model were expected. In order to gain more insight for the reasons of the observed spatial distribution for the present study we decided to split infant mortality into six groups defined by the underlying cause of death and to include the births of four more years so that now the study spans the time period of 1984 to 2006.

## Methods

Analyses were performed step-wise: 1) Estimation of expected counts of infant deaths for each Austrian administrative district using logistic regression models and individual birth certificates and 2) univariate and multivariate modelling of indirectly standardized mortality rates (SMR's) using Markov Chain Monte Carlo (MCMC) methods in Winbugs [4] based on observed and expected counts.

### Data

We analyzed 1,911,625 live births in 99 administrative districts, recorded between 1984 and 2006 in the Birth Certificate Registry managed by Statistics Austria [5]. Inclusion criteria for the data set were that the infants had been born as singletons between the 24^th ^and 44^th ^week of gestation, and to mothers between 13 to 50 years of age. Second or later born children were included if the period to the time of birth of a previous sibling was at least 40 weeks.

From birth certificates we extracted the following variables:

a) data on the newborn:

survival status after one year, year of birth, gestational age, birth weight, length at birth, sex;

b) data on the mother:

age, level of education, marital status, time interval from previous birth, parity, place of living (administrative district), means of subsistence, citizenship.

Two adjacent districts were joined because of the small number of their deaths recorded, so that a total of 98 districts entered into analysis.

Infant mortality (within the first year of life) was split up into six groups defined by the underlying cause of death. The following groups were formed: a) "Infections, respiratory diseases"; b) "Peripartal problems"; c) "Immaturity"; d) "Malformations"; e) "Sudden Infant Death Syndrom (SIDS)"; f) "All other". The definition of the groups by ICD-9 and ICD-10 codes was done by clinical experience [see Additional file 1].

### Calculation of expected counts

For each administrative district we calculated expected counts of infant deaths by means of logistic regression models using individual data in SAS [6]. All variables listed above were included except the administrative district. Based on the estimated regression model expected counts were calculated by summing up predicted probabilities for death by administrative district. This procedure was done for overall infant mortality including all six causes-of-death groups as well as for each of the six single groups. As the focus of this study is the description of the six group-specific spatial distributions, the results of the logistic regression models are not shown. For a detailed description of the effects of the covariates on overall infant mortality we refer to Waldhoer, et al., 2006 [3].

### Univariate and multivariate modelling of SMR's

For the univariate estimation of SMR by group of cause of death we used the conditional autoregressive model (CAR) also known as BYM or convolution model introduced by Besag et al. [7]. This model assumes, that the observed number of counts in spatial unit i is Poisson distributed with expectation μ_i_. Log(μ_i_) is assumed to be a sum of a spatially structured and unstructured random error S and U, respectively. S describes the information which is common to neighbouring units due to the spatial distribution of the underlying common risk factors. U stands for the spatially unstructured heterogeneity which may not be explained by factors in the model. This kind of model, also often called convolution model, is frequently used in spatial epidemiology. One reason for its popularity is its straightforward estimation by MCMC techniques e.g. in Winbugs [4].

The estimation of univariate CAR models for each cause-of-death group accounts for spatial autocorrelation of SMR's within the groups but ignores the fact that the 6 group specific spatial distributions may share information which could be used for a more precise estimation of the SMR's. In recent years some authors concentrated on the simultaneous analysis of two or more spatial distributions [8-10]. We used two different approaches, a multivariate CAR model and a shared component model, which also can be realized using Winbugs. In a multivariate CAR model (see e.g. Winbugs, Geobugs manual example 7) the univariate Gaussian conditional distribution for the random error terms S is replaced by a multivariate conditional distribution for the six random errors S_1_-S_6 _as in our case. This multivariate conditional distribution has a variance-covariance matrix with diagonal elements representing the conditional variances of the S_i_'s and off-diagonal elements representing the covariance between the S_i_'s.

Knorr-Held and Best [10] propose a shared component model where the spatial variation of two diseases is partitioned into a shared component and two-disease specific components (see e.g. Winbugs, Geobugs Manual example 8). Each of both components itself is modelled as a sum of a structured and unstructured random error. In our study we defined the expected number of deaths μ_ij _for the i-th district and k-th group as log(*μ*_*ik*_) = log(*E*_*ik*_) + *α*_*k *_+ *δ*_k _* *ϕ*_*i *_+ *ψ*_*ik*_

E_ik _is the expected number based on the logistic regression adjusted for the covariates, α_k _is a group specific intercept, δ_k _represents the strength with which the shared random error ϕ_i _determines the rate of the k-th group and Ψ_ik _is the k-th group specific random error of the i-th district. Both ϕ and ψ are then partitioned into a structured and unstructured random error. In order to make the model identifiable, the sum of the log(δ_k_) is set to zero.

The Deviance Information Criterion (DIC) [4] was used for model comparison. Moran's spatial autocorrelation coefficient I [11] was used for testing for a non-random uniform spatial distribution.

## Results

In Table 1 the number of deaths pertaining to each of the six groups can be seen. The group "malformations" contributes most, while "infections and respiratory diseases" contributes least to the number of deaths.

**Table 1:** Number of newborns alive and deceased by groups of cause of death

Group	Numbers	Percent
Alive	1,900,891	99.44
Infections, respiratory diseases	501	0.03
Peripartal problems	1,832	0.10
Immaturity	2,312	0.12
Malformation	3,557	0.19
SIDS	1,578	0.08
All others	954	0.05

Sum	1,911,625	100

### Overall infant mortality

**Figure 2:**
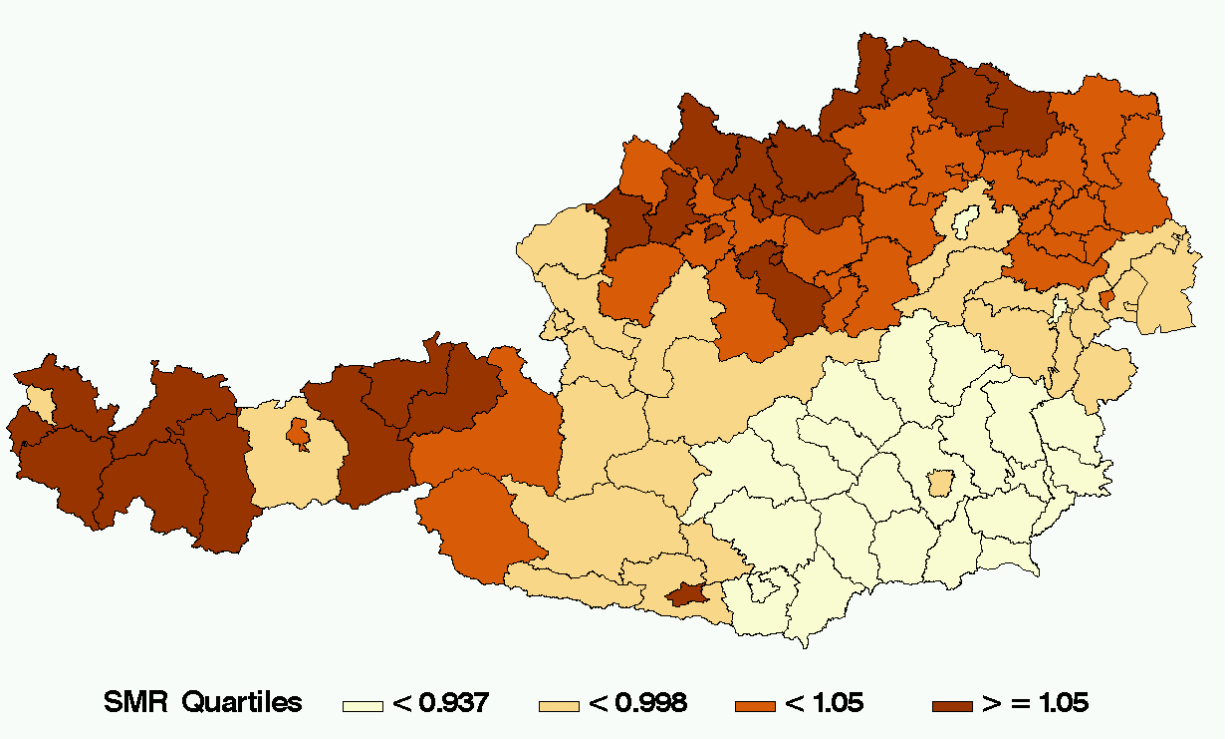
The spatial distribution of SMR's for overall infant mortality.

Figure 2 shows the spatial distribution of the SMR's of overall infant mortality (i.e. all six groups combined). In the South-East of Austria the risk of death within the first year of life clearly is lower than in the rest of Austria. The range of the SMR's is 0.83 to 1.21 showing differences in infant mortality of up to 45% within Austria.

### Mortality by cause of death estimated by a multivariate CAR model

**Figure 3:**
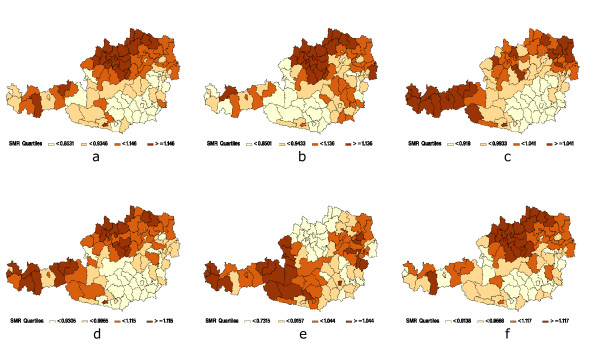
The spatial distribution of SMR's for infant mortality by groups of cause of death. a) "Infections, respiratory diseases"; b) "Peripartal problems"; c) "Immaturity"; d) "Malformations"; e) "Sudden Infant Death Syndrom (SIDS)"; f) "All other".

Figure 3 shows the spatial distributions for the six groups based on the multivariate CAR model. The groups "infections, respiratory diseases", "immaturity", "malformations" and "all other" exhibit a similar spatial distribution with clearly reduced SMR's in the South-East of Austria. In the group "peripartal problems" the area of reduced risk is shifted towards the West. "SIDS" has a different spatial distribution inasmuch as the area of lowest risk is clearly in the North of Austria.

In Table 2 the correlation coefficients between the structured components S of the six groups along with their 95%-credibility intervals are shown. All correlations are positive apart from the associations with "SIDS". Eight out of 15 credibility intervals do not cover zero and two intervals overlap zero to just a very small amount so that only five of the 15 correlations are left which show an effect just by chance. The associations are strong with values going up to 0.83 and therefore pointing to similar spatial distribution. Correlations with group "SIDS" are all non-positive pointing to a different spatial distribution of "SIDS" than the rest.

**Table 2:** Correlation between spatially structured components S of the multivariate CAR model

Group	Peripartal Problems	Immaturity	Malformations	SIDS	All others
Infections, respiratory Diseases	.77 (.18,.97)	.47 (-.26,.89)	.69 (.17,.94)	-.58 (-0.95,-0.1)	.83 (.47,.97)
Peripartal Problems		.32 (-.45,.82)	.53 (-.004,.88)	-.84 (-.97,-.58)	.82 (.41,.97)
Immaturity			.60 (-.03,.91)	.00 (-.64,.68)	.44 (-.28,.87)
Malformations				-.23 (-0.74,0.33)	.66 (.15,.93)
SIDS					-.64 (-.94,-.05)

### Mortality by cause of death estimated by a shared component model

**Figure 4:**
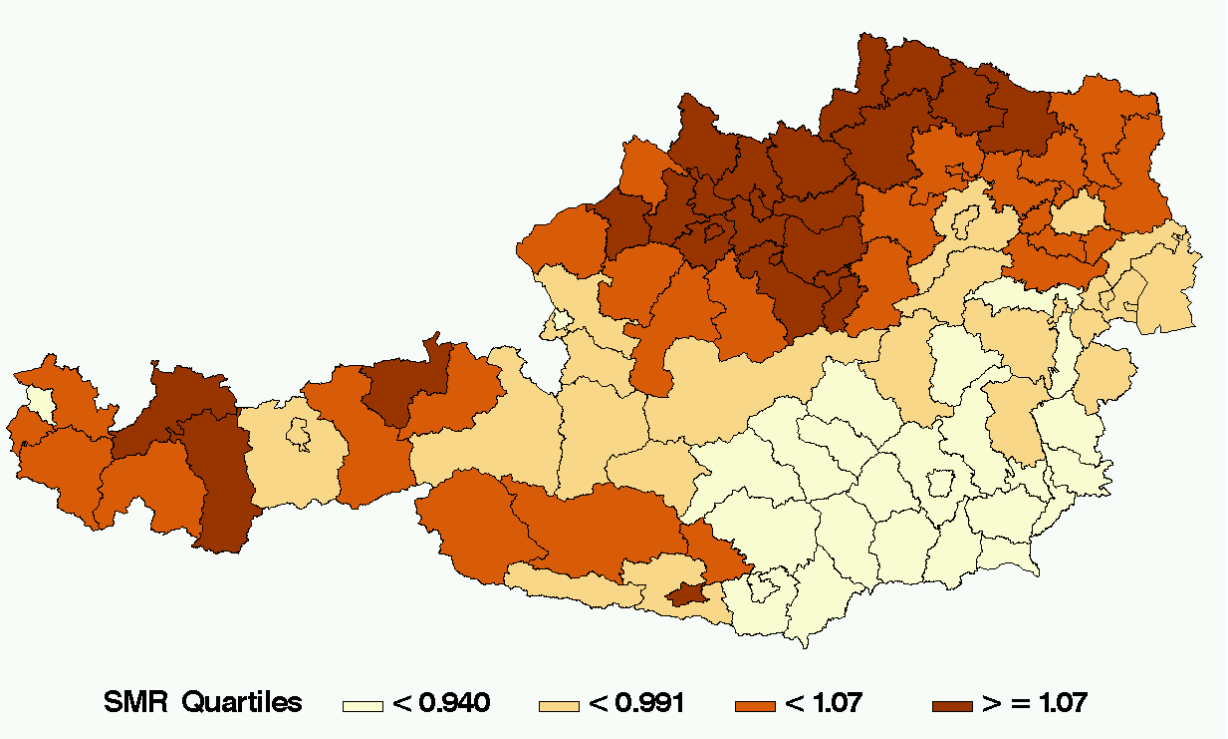
The spatial distribution of the shared component model.

Figure 4 shows the spatial distribution of the shared component ϕ. This shared component may be interpreted similar to a mean of all six spatial distributions while their importance for group k is described by the weight δ_k_. Unsurprisingly, this spatial distribution resembles the map of overall infant mortality strongly. The values of δ_k _in table 3 show that the groups "infections, respiratory diseases" and "all other" are strongly weighted, whereas "immaturity" and "SIDS" are least weighted with the shared component distribution. All credibility intervals are wide.

**Table 3:** Posterior distribution (mean) of weights δ and fraction shared by group for the shared component model and 2.5%, 97.5% credibility intervals

Group	mean δ	2.50%	97.50%	mean Fraction shared	2.50%	97.50%
Infections, respiratory diseases	2.17	0.97	3.39	0.76	0.07	0.99
Peripartal Problems	1.06	0.50	1.90	0.24	0.03	0.67
Immaturity	0.66	0.38	1.05	0.40	0.10	0.83
Malformations	0.90	0.54	1.37	0.67	0.27	0.95
SIDS	0.62	0.32	1.05	0.08	0.03	0.16
All others	1.51	0.80	2.36	0.85	0.40	0.99

**Figure 5:**
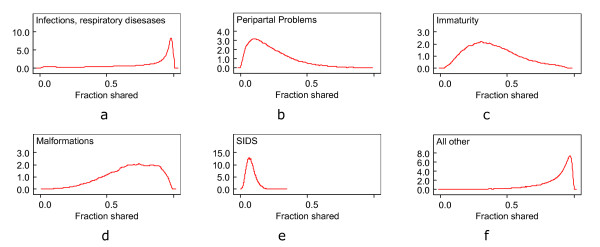
The posterior distribution of the fraction of shared variability by groups of cause of death.

The ranking of the fractions of the total between-area variation in risk is in accordance to the ranking of the δ_k _(Table 3). This fraction is the ratio of the empirical variance of the shared component, divided by the sum of the variance of shared and specific components. Clearly the most variability explained by the shared component occurs in the groups "infections, respiratory diseases" and "all other", the least fraction is explained in the group "SIDS". Credibility intervals for the fractions shared are very wide (Figure 5). Only the group "SIDS" exhibits a narrow interval which means that this group obviously does not have much in common with all other groups and therefore has a different spatial distribution.

### Comparison of models

The DIC value for the univariate model is 3198.8, compared to a DIC of 3169.2 (multivariate CAR model) and 3160.6 (shared component model). Having the smallest DIC value, the shared component model seems to fit the data best.

All spatial autocorrelation coefficients (Moran's I) are significant except for group 6 and range between 0.025 and 0.421 pointing to spatial autocorrelation and non-homogeneous risks.

## Discussion

In a recent paper [3] we were able to show that in the last 2 decades infant mortality was not randomly spatially distributed in Austria. In this present study including four additional years we were able to show that the spatial distribution of mortality within first year of life depends on the cause of death. To our knowledge such remarkable non-random spatial distributions have not been described up to now in a small, well developed country with homogeneously spread high-quality medical care.

Inequalities in quality of medical care within Austria should especially be perceivable in the groups "Infections, respiratory diseases", "Peripartal problems" and "Immaturity" [12-14]. Because of the observed non-random geographical distribution in these groups inequalities in quality of medical care could be reckoned as the underlying cause. However, this hypothesis contradicts the spatial distribution of the groups "Malformations" and "All other". These groups primarily comprise of diseases of which the outcome only weakly depends on quality of medical care as e.g. malformations, tumours, accidents and metabolic diseases and therefore should be distributed randomly in Austria.

The geographical distribution of SIDS nearly is inversely related to the other groups. Among other things, SIDS has been associated with prone and side positions for infant sleep, smoke exposure, soft bedding and bedding surfaces, overheating, use of pacifiers at sleeping time and room sharing without bed sharing [15]. Apart from these risk factors which are not available for our study, the regional distribution of SIDS may also depend on the quality of diagnosis which itself is linked to autopsy. In our study autopsy rates reached a high value of 80.4% but nevertheless autopsy rates also are non-randomly distributed within Austria [16] and therefore may influence the spatial distribution of SIDS. We intend to examine the reasons for the observed deviant regional distribution of SIDS in the future.

Infant mortality depends on many risk factors as e.g. social status and age of mother, gestational age, etc[3]. Many of these factors have been included in our models so that the observed regional patterns of SMR's are not dependent on the spatial distribution of these risk factors within Austria.

This study differs from many other studies dealing with spatial epidemiology as we used individual-based data and therefore avoided the well known ecological regression bias [17]. The size of the data base (1.9 million observations) prohibited direct model estimation with spatially structured and unstructured random errors because to our knowledge all available statistical software packages are uncapable to handle such large data sets for these kinds of models. Therefore the effect of 13 variables which have been shown to have a significant effect on infant mortality [3] was modelled by logistic regression models without random effects. Subsequently, the spatial structure was analyzed via observed and estimated deaths per district in WinBugs by means of univariate and multivariate based models including spatially structured and unstructured random errors.

The results showed that the estimated SMR's to some extent differ in their absolute values but the resulting choropleth maps using quartiles of SMR's do not and thus maintain the underlying spatial structure. In our opinion these similarities of the six maps based on 3 different estimation methods comes primarily from two facts: Firstly, the expected numbers of counts are large and therewith random variability does not superimpose the underlying spatial effect to a large extent. Secondly, the spatial effect of infant mortality is strong, even when mortality is split up into groups of causes of death.

In this study we used multivariate models analysing six spatial distributions simultaneously since it was to be expected that the spatial distributions are similar due to underlying covariates. The purpose of these kinds of models for disease mapping is borrowing strength for SMR estimation from spatial distributions of other diseases in addition to borrowing strength from neighbouring spatial units of the same disease [10]. This leads to shrinking mortality rates to a global mean based on the different group specific spatial distributions as well as smoothing of rates within disease groups. Consequently, this multivariate approach may produce more precise estimates than their univariate counterparts do.

The use of the multivariate CAR model specifically gives insight into pairwise associations by providing correlation coefficients between groups by cause of death. The CAR model may be contrasted with the shared component model which estimates something similar to a grand mean which apart from δ_k _is common to all disease groups. The relevance of this shared component may then be quantified by the fraction of variability explained by the shared component. This approach concentrates on the shared component and therefore may be useful when searching for the spatial distribution of underlying risk factors.

The results of our three different models are very similar in terms of the resulting disease maps so that a decision which model is to be preferred is not clear. However, the ranking of the DIC values shows that the model estimating six univariate BYM models has the largest DIC value and therefore seems to be less preferable. The multivariate CAR model exhibits a distinct lower DIC value than the univariate BYM model but a larger value than the shared component model. Apart from the DIC value, the choice between the multivariate CAR and the shared component model may be based on whether ones interest lies in pairwise associations or in detecting a spatial component common to all spatial disease distributions.

In our study we observed different non-random spatial distributions of infant mortality rates grouped by cause of death. The models were based on individual data and therefore excluded the possibility of ecological regression bias. In order to propagate the usefulness of two new methods, we used two estimation methods which have rarely been used in practice because of their recency. The results show that in our study the estimation method does not change the spatial distribution substantially.

The results of this as well as our previous study [3] show that the spatial distribution of the independent variables in the logistic regression model, like gestational age, age of mother etc. cannot explain the observed spatial disparities in infant mortality in Austria.

In order to gain more insight into the underlying causes for these disparities additional studies covering larger data sets are necessary. Adding ecological data based on the administrative district level requires rather limited efforts but is prone to the ecological regression bias and may thus only serve as a hypothesis generating approach. Certainly more efficient in terms of causal interpretations would be the addition of individual data which can be gathered in a retrospective or prospective way. Since retrospective or prospective acquisition of data for the total population of all newborns is not affordable, one of two feasible approaches would be a retrospective case-control study based on hospital based records comparing data from deceased and surviving newborns. Though gathering clinical data this way seems to be feasible, acquisition of further family related data (e.g. life style of parents etc.) could result in selection bias related problems since the response rate as well as the distribution of variables may differ between parents of deceased and surviving newborns. The second choice would be a prospective cohort study in which a group of newborns are followed to the end of the first year of life. This would allow the inclusion of new interesting variables which may be gathered soon after birth for all newborns. However, this would require a very large number of newborns (n ~200.000) because of the small infant mortality rate and therewith a 1-year follow-up of all Austrian newborns for about 3 consecutive years being not affordable in practice.

## Conclusion

In spite of a highly developed health care system we observed a clear spatial disparity in infant mortality in Austria. This is surprising since infant mortality is a sensitive parameter of health care systems and one should expect great efforts on the part of health policy to compensate for these inequalities. We were able to show that official birth certificates containing only limited clinical data give valuable but not sufficient information to explain the observed disparities. We conclude, that further studies are needed which involve additional individual as well as clinical data in order to explain the observed disparities in mortality rates in Austria.

## Competing interests

The authors declare that they have no competing interests.

## Authors' contributions

TW had the main responsibility for carrying out the study and writing the manuscript.

MW participated in conceiving the study and writing the medical part of the manuscript.

HH participated in conceiving the study and writing the manuscript.

All authors read and approved the final manuscript.

## Supplementary Material

Additional file 1 Grouping_ICD_Codes_9_10. Grouping of deceased newborns by ICD-9 Codes until 2001 and ICD-10 Codes after 2001. Click here for file
